# Paving the way for the future of child and adolescent mental health in Japan

**DOI:** 10.1080/17571472.2018.1483002

**Published:** 2018-06-11

**Authors:** Mari Sakano, Natasha Snowden

**Affiliations:** a Rainbow Forest Clinic, Tottori, Japan; b Institute of Psychiatry, Psychology & Neuroscience, King’s College London, London, UK

**Keywords:** Japan, mental health services, child psychiatry, child psychology, CAMH

## Abstract

Japan’s healthcare system is primarily focused on general care, and psychiatric services are mainly concerned with institutional solutions to serious mental health issues. As a result, child and adolescent mental health services (CAMHS) have been slow to develop, and there is a limited evidence base to guide treatment. As such, providing treatment to this population is a challenging endeavour. This landscape piece aims to illustrate the current state of child and adolescent services in Japan and provides a description of a recently opened private clinic for children with mental health conditions in Kuroyashi, a city in the Tottori prefecture. The vignette describes steps taken by the clinic’s director to overcome systemic challenges and increase access to mental health services to children and adolescents.

**WHY THIS MATTERS TO ME**

After 13 years of practice in both paediatrics and psychiatry, I knew that I needed to advance my skills in order to work with children with severe mental health difficulties. I decided that King’s College London and the Institute of Psychiatry, Psychology and Neuroscience (IoPPN) would equip me with an excellent foundation of knowledge in child and adolescent mental health. Together with my two children, we set off for a year in London in July 2016.

The year I spent at the IoPPN was both inspiring and enlightening. While studying, I began to understand that much of the protocol and procedure in my practice did not have a rigorous evidence base. I also came to be impressed by the number of psychotherapists employed in community Child and Adolescent Mental Health Services (CAMHS) and in hospitals; they are almost non-existent at home. Seeing the importance of these functions, I decided that I wanted to play a part in transforming child and adolescent mental health care in Japan. Though I knew it would be an uphill climb, I set forward on the challenge upon my return home in October 2017.

I am delighted to share the experience of practicing in Japan, and subsequently opening my own clinic, with the journal’s audience. Although Japan is known as a developed nation, it is still developing in terms of child mental health care. However, I am determined and optimistic; it is my hope that this case study can serve to educate and inspire both students and practitioners around the world.

**KEY MESSAGE**

Lessons from a case study in CAMHS in Japan

## The context of the case study

Japan’s population is one of the oldest in the world. Children and young people aged 18 years and under account for 16.2% of the population [1], compared to 22.3% in the UK [2]. As such, the Japanese social welfare system is largely focused on geriatric care rather than child and adolescent care. Although Japan has the third highest GDP in the world, public spending on family benefits constitutes only 1.74% of the total GDP [3].

All Japanese citizens are entitled to health insurance coverage by the government and cannot be denied coverage or access to health care. One of the benefits of Japan’s health system is that it guarantees universal access. That is, the GP system is not restrictive, and people can see any doctor at any hospital or clinic in the country. A referral is not always mandatory to see a specialist, though patients who come without a referral pay higher fees. Although this increases choice, it means that there are significant demands on doctors’ time. Hospitals are generally overcrowded, which is mitigated by longer working hours for doctors. Likewise, patients see doctors for short appointments. Government statistics showed that 67.7% of patients could have only less than ten minutes to see a doctor at the outpatient clinics [4]. Access to psychiatrists is no exception.

Despite having one of the highest suicide rates in the world, prevalence of mental health conditions is not yet certain in Japan. In 2014, 1.1 million people saw a doctor due to an affective disorder [5]. This suggests that the demand for mental healthcare in Japan may well be high. However, psychiatric care in Japan has primarily developed around the need to treat people through institutionalised care and pharmacological therapy. Japan does have more psychiatric care beds than any other OECD country (2.65 per 1,000 population in Japan vs 0.42 in the UK [6]). Also, the average length of psychiatric hospital stay is 269.9 days [7], comparing to 37.7 days in the UK [8]. This has led to the underdevelopment of psychotherapy as a treatment for mental illnesses, which has reduced the opportunity for patients to access care that is appropriate to their needs. The problem has also been exacerbated by the lack of a nationally recognised qualification for psychotherapists. This lack means that psychotherapy, at present administered by clinical psychologists, has not been covered by health insurance. However, in November 2018 a nationally recognised qualification – that of ‘Certified Psychologist’ will be passed into law.

CAMHS sits at the cross-section of two underfunded and underdeveloped areas in Japanese social and health policy – mental health and childcare. Childcare receives scant attention in Japanese politics. Although the Child Welfare Act was enacted in 1947 for the protection of children, like psychiatric care, the primary focus of social care has been institutionalisation. The vast majority of Japan’s 45,000 children in care are in institutions, while only 11.5% of them live with foster parents [9]. Adoption is even less common and correspondingly life outcomes for children in care are particularly bad. Only 24.0% of children in care receive higher education compared to 74.1% of adolescents generally [9].

The provision of mental healthcare for children is also inadequate, in a country where children and adolescents make up 16.2% of the population. Psychiatrists qualified to treat children and adolescents make up just 0.025% of the entire psychiatrists’ population who practice in Japan [10]. There is also no formal training curriculum for child and adolescent mental health care specialists.

Research is also under-developed compared to other developed nations. There are no national level observational studies. Apart from medication for ADHD, most other psychiatric medications for children are not formally covered by health insurance due to a lack of clinical studies from Japan; an essential requirement for coverage by insurance.

## A case study

Kurayoshi is a small city in the south west of Japan, with a mix of historical sites, shops, restaurants and a large number of farms. It sits within Tottori prefecture, the least populous of all 47 of Japan’s prefectures with around 590,000 people living in the region. There are two residential children’s homes for disabled or abused children, and two women’s shelters for victims of domestic violence. Before leaving to study at King’s College London, I was the only child and adolescent psychiatrist at the city’s psychiatric hospital. However, after I left, no one replaced me in my role. Following my time in London, I returned to Kurayoshi in October 2017 and began the work to open my own private clinic – ‘Rainbow Forest Clinic’. We officially opened our doors in 2018.

The clinic provides various kinds of psychotherapy. The clinic struggles with financial issues which constrains the time available for treatment. This problem is exacerbated by the fact that it is difficult to hire psychologists, as psychotherapy isn’t covered by health insurance. This means that patients often turn to psychiatrists to prescribe medication. To face these challenges, I am in the process of setting up a day care centre separated from the psychiatric clinic itself, which will provide psychotherapy to children and young people aged 6–18. As public funding is available for them, I anticipate being able to offer affordable care for them. I also plan to introduce various forms of psychotherapy including dialectical behaviour therapy, mentalisation based therapy and parenting programs.

Additionally, I plan to develop a child and adolescent mental healthcare network to provide organised care. As Kurayoshi is a small rural city, many people know each other well and are keen to learn from one another. As I am often invited to speak on the subject of mental health, I anticipate that I will be able to collaborate with schools, childcare facilities, city councils and other similar institutions to establish primary care mechanisms to support mental health. This might introduce screening systems and early interventions in schools, as well as parenting support in the community.

Finally, I will work with local policymakers to encourage them to provide sufficient funding for CAMHS in the prefecture. This might include supplying educational resources, online tools and information, easy access to consultants for teachers, and financial and psychological support for foster parents and adoptive parents. Effecting change at a national level would be particularly challenging at such an early stage. However, as every prefecture in Japan has its own local legislature, I think that progress in Tottori is a realistic ambition.

The Rainbow Forest Clinic has only just opened, and our challenging journey has just begun. Since returning to Japan in October 2017, my personal and professional life has been totally consumed by opening and running the clinic as well as the hectic preparations for opening the day care centre. Due to this, I have yet to produce any data to demonstrate the effect of my actions on the lives of children. However, I am certain that my commitment to overcome the challenges ahead of me will allow me to provide valuable care to vulnerable children. Improving their lives and inspiring hope is my ultimate goal and their happiness is the data-set I care most about.

My time at King’s underlined the central importance of research and evidence in psychotherapy, and exposed the fact that my previous practice in Japan had not been based on robust evidence. There is a scarcity of good data and evidence in Japan, so my collection of primary data is a vital component to improve the quality of research in the country. I plan to create and manage the database at my clinic and will share access to it with other doctors conducting research.

## Disclosure statement

No potential conflict of interest was reported by the authors.
